# Identification of angry faces in the attentional blink

**DOI:** 10.1080/02699930701774218

**Published:** 2008-02-06

**Authors:** Frances A. Maratos, Karin Mogg, Brendan P. Bradley

**Affiliations:** University of Southampton, Southampton, UK

## Abstract

According to cognitive and neural theories of emotion, attentional processing of innate threat stimuli, such as angry facial expressions, is prioritised over neutral stimuli. To test this hypothesis, the present study used a modified version of the rapid serial visual presentation (RSVP) paradigm to investigate the effect of emotional face stimuli on the attentional blink (AB). The target stimuli were schematic faces which depicted threatening (angry), positive or neutral facial expressions. Results showed that performance accuracy was enhanced (i.e., the AB was reduced) on trials in which the second target was an angry face, rather than a neutral face. Results extend previous research by demonstrating that angry faces reduce the AB, and that this effect is found for schematic facial expressions. These findings further support the proposal that, when there is competition for attentional resources, threat stimuli are given higher priority in processing compared with non-threatening stimuli.

## INTRODUCTION

Cognitive and neural models of fear and threat processing propose that there are specialised mechanisms that are responsible for the enhanced processing of threat-related information (e.g., Davis & Whalen, 2001; LeDoux, 1996; Mogg & Bradley, 1998; Öhman, 1996; Pessoa, 2005). Such models suggest that attentional resources are preferentially allocated to threat-related cues, relative to other types of information (e.g., non-emotional stimuli), and that this selective processing should be particularly apparent for biologically prepared threat stimuli, which have evolutionary significance, such as angry faces, snakes and spiders (LeDoux, 1996; Öhman, 1996). The ability to process such information efficiently poses several survival advantages. For example, speed is important when coping with threat-related stimuli such as fear-relevant animals (e.g., snakes) or negative emotional expressions (e.g., angry face); fast identification of such stimuli allows early activation of defence systems (LeDoux, 1996; Öhman, 1996). Thus, it is no surprise that recent behavioural and neuroimaging research has focused on how emotional stimuli influence information processing.

A main theme emerging from this research is that the processing of threat stimuli is prioritised relative to non-threat stimuli. Indeed, it has been proposed that threat stimuli capture and hold attention in a manner unlike that of non-emotional stimuli (e.g., Öhman, 1996; Öhman, Lundqvist, & Esteves, 2001), have a privileged processing status (Vuilleumier & Schwartz, 2001) and, controversially, can be processed independently of top-down attention (Öhman, 2002; but see also Pessoa, Kastner, & Ungerlieder, 2002).

One paradigm that has been used to investigate attentional processing of emotional information is the rapid serial visual presentation (RSVP) task, which assesses a cognitive phenomenon known as the “*attentional blink*” (AB). In a typical RSVP study of the AB, two targets are presented in a stream of distractor stimuli. If these targets are presented in quick succession, accurate report of the second target is impaired when it is presented 200–400 ms (or 2–3 items) after the first target (e.g., Kranczioch, Debener, Schwarzbach, Goebel, & Engel, 2005; Shapiro, Arnell, & Raymond, 1997a). This performance decrement, or AB, is thought to reflect competition between different stimuli for attentional resources. According to cognitive theories of the AB, the effect occurs at an early stage of processing within the cognitive system where there are limited-capacity processing resources (see Shapiro et al., 1997a, for a review).

Interestingly, when the second target is a motivationally salient or emotional stimulus, the AB is much reduced. For example, Shapiro, Caldwell, and Sorensen (1997b) found that the AB was abolished when the second target stimulus in an RSVP stream was the participant's own name. Other studies, which have used emotional words (or Chinese ideographs, which vary in emotional valence) as target stimuli, have found that the AB is reduced when the second target stimulus is a negative or aversive stimulus, rather than a neutral stimulus (e.g., Anderson, 2005; Anderson & Phelps, 2001; Ogawa & Suzuki, 2004). Thus, this research suggests that emotional stimuli, such as negative words, have a special attentional status and are more readily detected than neutral stimuli (see also Barnard, Ramponi, Battye, & Mackintosh, 2005; Most, Chun, Widders, & Zald, 2005, for additional evidence from RSVP tasks of attentional effects of emotional stimuli).

However, Anderson (2005) noted that RSVP studies using emotional words as stimuli do not directly address predictions from cognitive and neural models, which propose that attentional prioritisation of threat cues should be most apparent for special classes of biologically prepared stimuli, such as angry faces (Öhman, 1996). Thus, there are strong theoretical grounds for using face, rather than word, stimuli in order to investigate the cognitive mechanisms underlying selective processing of threat. The face is a socially and biologically significant stimulus as well as an important index of emotional information. Moreover, evidence from other paradigms, such as visual search, or visual cueing tasks using briefly presented masked face stimuli, indicates that angry faces are processed preferentially over neutral faces (e.g., Calvo, Avero, & Lundqvist, 2006; Mogg & Bradley, 1999; Öhman et al., 2001), although controversy exists over specific methodological issues relating to such paradigms (Pessoa, 2005). Two recent studies examined the effect of fearful faces on the AB, as fearful expressions are threat-relevant, given that they signal threat in the environment, although they are not threatening per se. Fox, Russo, and Georgiou (2005) found that high anxious individuals showed a reduced AB for fearful faces, relative to happy faces, whereas Milders, Sahraie, Logan, and Donnellon (2006) found that the AB was reduced by fearful faces, relative to both happy and neutral faces, in unselected adult participants. Both studies used photographic face stimuli. However, some researchers have advocated the use of schematic face stimuli in preference to “real” faces, because schematic faces are less prone to potential confounds associated with difficulty in controlling low-level perceptual features and familiarity (Fox et al., 2000; Juth, Lundqvist, Karlsson, & Öhman, 2005; Öhman et al., 2001).

Consequently, in this study we extended previous research by using a schematic face version of the RSVP paradigm, which included emotional faces as target stimuli, to assess the depth and temporal resolution of the AB to threatening, positive and neutral stimuli. If threatening stimuli are processed preferentially, then an angry face should result in a reduction of the AB phenomenon when the face appears as the second target. Specifically, our main hypothesis was as follows: if threatening faces have a privileged processing status, then participants' accuracy in target-identification will be enhanced if the second target in the RSVP stream is a threatening face, rather than a neutral or positive face. This effect should be evident when there is a short interval between the two targets (approx. 200–400 ms), which corresponds to the time window of the AB.

## METHOD

### Participants

These were 23 students (14 female; mean age = 25.6 years, *SD* = 3.7 years) from the University of Southampton. The selection criteria were (i) normal or corrected-to-normal vision and (ii) acceptable level of accuracy in detecting single target stimuli, in both the screening and main RSVP tasks (see procedure for details). The latter criterion was adopted because if participants could not detect a single target reliably in a RSVP stream, their results from critical trials with two targets would be difficult to interpret. Two participants were excluded due to below-criterion performance on the screening task and a third participant was excluded because of poor accuracy on single-target control trials (below two *SDs* of the sample mean). Thus, data from 20 individuals (13 females; mean age = 25.6 year, *SD* = 3.5 years) were analysed. Participants who completed the experiment received £9 payment.

### Stimuli

**Figure 1:**
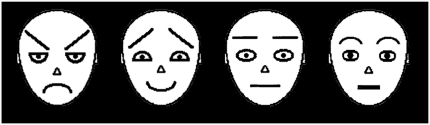
Illustration of schematic face stimuli displaying threat, positive and two neutral facial expressions.

**Figure 2:**
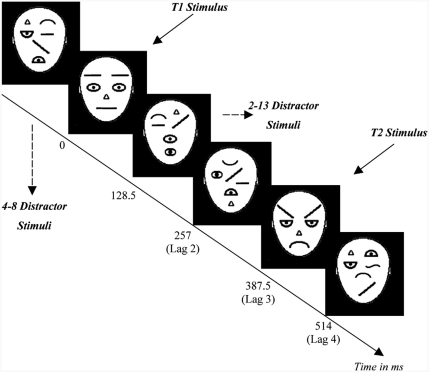
An example of a double-target trial in which T1 was a neutral face (N1) and T2 was a threat face.

Four schematic faces were used as target stimuli: a threat face, a positive face and two neutral faces (see Figure 1). The two emotional faces and one of the neutral faces were the same as those used by Öhman et al. (2001): the threat face was their “angry” exemplar and the positive face was their “friendly” exemplar. The latter is referred to here as a “positive” face, as it has been described previously as both a “friendly” face and a “happy” face (Calvo & Esteves, 2005; Juth et al., 2005). All four face stimuli differed with respect to three main features; eyebrow, eye and mouth shape (e.g., when comparing the threat and positive faces, the mouth and eyes were inverted and the eyebrows switched). Two non-identical neutral faces were used to minimise potential effects of repetition blindness (i.e., reduced ability to detect the second of two identical items in a RSVP stream; Kanwisher, 1987). There were also 30 different distractor stimuli, which comprised the key features of each face stimulus in random positions and orientations (see Figure 2 for examples of distractor stimuli). All stimuli subtended avisual angle of 5.7° × 7.5° and were displayed on a black background at a viewing distance of 50 cm. Stimulus presentation was controlled by Millisecond software (www.millisecond.com). Each stimulus was presented for 128.5 ms using a 70 Hz refresh rate (i.e., each image was displayed for nine screen refreshes at a 70 Hz refresh rate resulting in a display time of 128.5 ms; these durations were determined in pilot work and checked with an oscilloscope).

### Procedure

The experiment consisted of two tasks: a short screening RSVP task and the main RSVP task. All trials contained a RSVP stream of 20 stimuli. In the *screening* task, on each trial, a single target face stimulus, which was the threat face, the positive face or one of the two neutral faces, was presented within a stream of 19 distractor stimuli. The target face was always preceded by at least five and followed by at least two distractors. Thus, the target face (randomly chosen across stimulus types) could appear in one of the remaining 13 positions in the stream with equal frequency. Participants were required to press one of three response keys to indicate the expression of the target face. Selection criterion was achieved if an individual correctly identified the expression of the target faces on nine consecutive trials within a limit of up to 50 trials. The presentation of stimuli in the screening task was similar to that on single-target trials in the main RSVP task.

The *main* RSVP task consisted of one block of 10 practice trials and six blocks of 106 test trials (i.e., 636 test trials in total, which were presented in a single session). Test trials consisted of 156 (25%) single-target trials and 480 (75%) double-target trials. At the beginning of each trial a small circle was presented for 214 ms at the central fixation point. On double-target trials, after the central fixation stimulus, the stimulus events were as follows: an initial sequence of distractor stimuli (ranging from 4 to 8 consecutive stimuli on each trial), the first target (T1), another sequence of distractor stimuli (between 0 and 8 stimuli), the second target (T2), and then the remaining distractor stimuli (between 2 and 13 stimuli; see Figure 2). After each RSVP stream, participants were required to make two consecutive responses to indicate (i) whether one or two face stimuli had been presented (by pressing buttons labelled 1 or 2) and (ii) the emotional expression of the *last* face viewed (by pressing buttons labelled A, H or N to indicate whether the last face was angry, happy or neutral). Thus, participants were asked to detect T1, but not to identify its emotional content (N.B. semantic identification of T1 is not necessary to reveal the AB; Barnard et al., 2005).

The double-target trials of primary interest were those in which the T1 was a neutral face (either neutral1 or neutral2), and the T2 was a threat, positive or neutral face. This resulted in three main trial types (i.e., three levels of the within-subject independent variable of Trial Type), which depended on the emotional content of the T2:

1. Neutral T1–threat T2 (threat trials);
2. Neutral T1–positive T2 (positive trials); and
3. Neutral T1–neutral T2 (neutral trials).

On each trial, T1 and T2 were always different stimuli; i.e., if T1 was neutral2, T2 was neutral1, or vice-versa.

For each of these three main trial types, the number of intervening distractors between T1 and T2 varied; so that, on each trial, there could be none, one, two, three, four, five, seven or eight intervening distractor items between T1 and T2. The primary conditions of relevance to the hypothesis were those in which there was at least one intervening distractor between T1 and T2.^1^ These seven conditions represented differing levels of the within-subjects independent variable of Lag position (where “lag” refers to the serial position of T2 in the RSVP stream, *relative* to the T1 position). Each of the seven lag positions corresponded to a stimulus onset asynchrony (SOA) between T1 and T2 of 257 ms (Lag 2), 385.5 ms (Lag 3), 514 ms (Lag 4), 642.5 ms (Lag 5), 771 ms (Lag 6), 1028 ms (Lag 8) and 1156.5 ms (Lag 9). The design of the experiment was such that, for each of these lag conditions, there were ten trials for every T1–T2 Trial Type (i.e., threat, positive and neutral trials).

The single-target trials were the same as the double-target trials, with the exception that only one target was presented (i.e., the T1 was replaced by a distractor stimulus). Thus, the target stimulus on single-target trials was presented under the same conditions as the T2 on double-target trials (i.e., in all equivalent serial positions in the RSVP stream).

Finally, to ensure that not all T1 stimuli were neutral and that not all emotional stimuli were paired with a neutral stimulus, four further trial types were included: (1) threat T1–neutral T2 (80 trials); (2) positive T1–neutral T2 (80 trials); (3) threat T1–positive T2 (40 trials); and (4) positive T1–threat T2 (40 trials). For each of these trial types, the number of trials at each lag position was balanced so that there was an equal number of trials in each lag position, as described earlier for the experimental trials.

## RESULTS

### Single-target (control) trials

On single-target trials (with no T1 target), the mean percentage of trials on which both the number and type of target were correctly identified was 86%. An analysis of variance (ANOVA) of these correct responses was carried out with Face Type (threat, positive, neutral) and Serial Position (early, mid, late)^2^ as independent variables. This revealed only a significant main effect of face type, *F*(2, 38) = 5.62, *p* < .01, $\eta_{\text{p}}^{2}=.23$. Pair-wise Bonferroni corrected comparisons revealed poorer performance on trials with positive faces (*M* = 82%, *SD* = 16) compared with threat faces (*M* = 87%, *SD* = 13) and neutral faces (*M* = 89%, *SD* = 8). Of importance, the mean error rate for trials with threat targets did not significantly differ from those with neutral targets. A supplementary analysis was also carried out to compare performance accuracy for the two exemplars of neutral faces (neutral1 vs. neutral2); this analysis showed no significant results, *t*(19) = 0.66, *p* > .5, so the data from these two exemplars were collapsed into a single neutral face category.

### Double-target (experimental) trials

**Figure 3:**
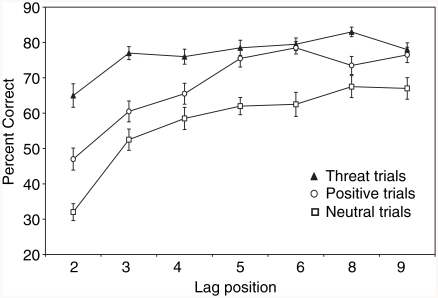
Mean percentage of correct answers (with standard error bars) on double-target trials; i.e., trials in which both the number of targets and expression of the second target were correctly identified. The T1 was a neutral face and the T2 was a threat, positive, or a different neutral face; i.e., trial type refers to emotional content of the T2 face.

Figure 3 shows the mean percentage of double-target trials with correct responses (i.e., trials where both the number and type of target were correctly identified); these data are illustrated as a function of Trial Type (threat, positive, neutral) and Lag (seven levels). An ANOVA of the percentage of correct responses, with Trial Type and lag as independent variables, revealed significant main effects of Trial Type, *F*(2, 38) = 10.22, *p* < .01, $\eta_{\text{p}}^{2}=.35$, and Lag, *F*(6, 114) = 16.12, *p* < .001, $\eta_{\text{p}}^{2}=.54$, and a significant interaction between the two, *F*(12, 228) = 1.93, *p* < .05, $\eta_{\text{p}}^{2}=.08$.

To clarify the interaction, a one-way Bonferroni-corrected ANOVA of the percentage of correct responses, with Trial Type (threat, positive, neutral) as the independent variable, was undertaken separately for each lag position. Results showed a significant difference in performance accuracy between the trial types at lag 2, *F*(2, 38) = 16.90, *p* < .001, $\eta_{\text{p}}^{2}=.47$, and lag 3, *F*(2, 38) = 8.97, *p* = .001, $\eta_{\text{p}}^{2}=.32$. To establish the cause of these differences, pair-wise Bonferroni corrected comparisons for threat, positive and neutral trial types were undertaken at both lag 2 and lag 3. These simple effects analyses revealed that at both lags, performance accuracy was significantly better on threat trials in comparison with neutral trials (lag 2: *p* < .001; lag 3: *p* < .01) and also in comparison with positive trials (lag 2: *p* < .05; lag 3: *p* < .01). Thus, when the T2 occurred within the 200–400 ms time window of the AB, there was greater accuracy in identifying the second target stimulus when it was a threat face, rather than a neutral or positive face. The pairwise comparisons further revealed that performance accuracy was significantly better on positive trials than on neutral trials at lag 2 (*p* < .05).

Finally, further analyses were conducted to address a supplementary question of whether or not a performance decrement (corresponding to an AB) occurred on trials with threat (T2) targets. As can be seen in Figure 3, performance accuracy on threat trials appeared to be worse when the threat T2 occurred shortly after the neutral T1, compared with when it occurred later in the RSVP stream. To evaluate this, performance accuracy on threat trials at lag 2 (which is within the temporal domain of the AB) was compared with mean performance accuracy on threat trials averaged across lags 5, 6, 8 and 9 (lags not within the temporal domain of the AB). Results showed that performance accuracy on trials with threat T2 targets was significantly worse at lag 2 than at later lags, *t*(19) = 2.38, *p* < .05.

## DISCUSSION

The present study used a schematic face version of the RSVP paradigm, which employed emotional and neutral faces as target stimuli, in order to test a key prediction from cognitive and neural models of emotion concerning the prioritisation of processing of threat information. In support of this prediction, the results indicated that participants' ability to identify target face stimuli in a RSVP stream of distractor stimuli was enhanced if the second target stimulus was a threat face, rather than a neutral face. This effect was only evident within the temporal domain of the AB, i.e., when the second target face was presented shortly after the first (neutral) target face stimulus. Specifically, on trials that had only one or two intervening items between T1 and T2 (which corresponded to T1–T2 SOAs of 257 and 386 ms, respectively), participants were significantly better at identifying targets when the second face was threatening rather than neutral in emotional content. These findings are compatible with theories of emotion which propose that there are specialised cognitive mechanisms which promote attentional processing of threat cues, such that these cues are prioritised over processing of other types of information (e.g., LeDoux, 1996; Öhman, 1996).

The results are also largely consistent with previous RSVP studies of the AB, which have used word (or ideographic) stimuli to examine selective processing of emotional information. As noted in the introduction, these studies have similarly found that the AB is attenuated when the second target is negative (or high in emotional arousal), rather than neutral in emotional content (e.g., Anderson, 2005; Ogawa & Suzuki, 2004). The present findings additionally complement the recent work of Fox et al. (2005) and Milders et al. (2006), who found evidence of a reduced AB for photographic representations of fearful faces. The results reported here not only indicate that this attentional effect extends to the processing of angry facial expressions, but also that it can be found for schematic faces, which have been proposed to provide a relatively pure representation of key features of emotional faces (Fox et al., 2000; Juth et al., 2005; Öhman et al., 2001). Indeed, as noted in the latter studies, a further advantage of schematic faces is that they allow the investigation of processing biases for emotional stimuli while controlling for potential confounds that are more difficult to control when using real-life faces (e.g., variation in physical features such as contrast, luminance, etc.; see Öhman et al., 2001, for further discussion of this issue). Thus, the current findings further suggest that the processing advantage observed for threatening faces in AB studies is due to the emotional meaning of the stimuli rather than image artefacts. However, the use of schematic faces is not without potential limitations, as, for example, there are few exemplars (e.g., only one angry face used here) and Horstmann, Borgstedt, and Heumann (2006) have suggested that both emotional and perceptual variables could contribute to their effects. Thus, it seems essential for research to evaluate converging lines of evidence from studies using varied types of stimuli, including real-life and schematic representations of pictorial threat, as each methodology has its own advantages and disadvantages. It would also seem helpful to clarify whether certain features of facial expressions associated with anger (e.g., shape of eyebrows or mouth) are more readily identified in RSVP streams than those associated with neutral or positive expressions (Lundqvist & Öhman, 2005).

The present results indicated that the AB was reduced by threat faces, relative to neutral faces (at lags 2 and 3) and that this effect was not explained by a difference in the discriminability of these stimuli, given that no difference was found between the threat and neutral face types on single-target trials. The AB was also reduced by positive faces, relative to neutral faces (at lag 2), which is not easily explained by differences in stimulus discriminability, as there was no evidence from single-target trials to suggest that positive faces were easier to identify (indeed, the opposite was found, i.e., poorer identification of positive than neutral faces when presented alone in the RSVP stream). However, the finding of a larger AB for positive faces, relative to threat faces, is not entirely conclusive, given that positive faces were also harder to identify than threat faces on single-target trials. One important line for future research would be to investigate further the relative influences of stimulus valence and stimulus arousal on attention. The positive face stimulus used in the present study was selected by Öhman et al. (2001) to be the antithesis of the threatening (angry) face. Consequently, it was designed to be low on stimulus dimensions that are likely to be associated with arousal, including activity and potency (Lundqvist & Öhman, 2005). Thus, the positive-face stimulus may have differed from the threat and neutral faces not only in terms of emotional valence, but also in terms of its arousal-provoking properties. Recent research that has used verbal, rather than pictorial, stimuli as targets suggests that the arousal value of word stimuli is an important determinant of the extent of the AB (Anderson, 2005; Keil & Ihssen 2004). In order to investigate further the relative influences of stimulus valence and arousal on attentional mechanisms, it may be helpful to use real-life pictorial representations of emotional faces, because real-life faces not only have good ecological validity, but also a potentially wider range of emotional valence and arousal values for both threatening and positive facial expressions, relative to schematic faces.

Another interesting finding from the present study was that, although the AB on trials with threat T2 faces was attenuated relative to trials with neutral T2 faces, there was still evidence of a residual AB for threat faces. That is, the results indicated an impaired ability to identify the threat T2 when it appeared at a short lag (T1–T2 SOA of 257 ms) relative to when it appeared at later lags (T1–T2 SOAs ranging from 643–1157 ms). The pattern of means in Fox et al. (2005) and Milders et al. (2006) similarly suggest that the AB effect was attenuated, rather than eliminated, by fearful faces (i.e., identification of fearful T2 faces appeared to be poorer at early lags compared with later time lags). Thus, one research question that remains to be addressed is whether the AB effect would be abolished entirely when more aversive stimuli are used, such as real-life stimuli depicting intense anger.

Given that the present results support the hypothesis that threat stimuli are accorded higher priority (relative to neutral stimuli) when there is competition for processing resources, it is helpful to consider the mechanisms that may underlie such emotional influences on the AB. A dominant theoretical view is that performance decrements associated with the AB arise because the two targets (T1 and T2) and their immediately following distractor stimuli compete for access to a limited supply of processing resources (Shapiro et al., 1997a). Anderson (2005) recently reviewed several ways in which emotional stimuli might “win” in this competition. For example, the cognitive representations of emotional stimuli in short-term memory may persist longer than those of neutral stimuli, which would allow them an advantage in subsequent processing. Alternatively, threat cues could be processed automatically (LeDoux, 1996) and consequently place less demand on processing resources in order to reach awareness. Third, emotional stimuli may activate a network of neural structures, including the amygdala, prefrontal and sensory cortices, which in turn modulate attention and visuo-perceptual processing, resulting in enhancement of the subjective perceptual experience of threat cues and their ability to capture attention (e.g., Davis & Whalen, 2001; Vuilleumier, 2005).

Recent research has indicated that, within the time window of the AB, healthy individuals who have intact amygdalae are better at detecting aversive words than neutral words; whereas patients with left amygdala damage do not show this effect (Anderson & Phelps, 2001). Such findings are consistent with neurocognitive models that propose an intimate relationship between the neural mechanisms underlying emotional and attentional processing (e.g., Davis & Whalen, 2001; Vuilleumier, 2005). It would therefore be informative to extend research into the neural basis of attention to threat cues, by using RSVP paradigms with emotional faces as stimuli (as in the present study). For example, such research could examine whether the extent to which the AB is attenuated by threat faces (relative to neutral faces) is predicted by differences between the activation effects of threat and neutral faces within neural structures, such as the amygdala and visual cortices.

## Footnotes

1 The condition in which there was no distractor between T1 and T2 was not included in the analyses because the AB effect is often not found in this position (so-called “Lag-1 sparing”) and there is evidence that different mechanisms are responsible for Lag-1 sparing and the AB effect (Chun & Potter, 1995; Hommel & Akyurek, 2005; Shapiro et al., 1997a). As the aim of the study was to investigate the effect of emotional information on the AB, the main hypothesis concerned identification of T2 faces during the time window of the AB (i.e., 200–400 ms following T1) versus after the AB (>500 ms after T1).

2 Targets presented at serial positions 7–10, 11–14 and 15–18 formed the early, mid and late serial position levels, respectively. The data were collapsed from 12 positions into these three categories to reduce floor effects in the analyses.
